# Long-Term Follow-Up of Patients with Conjunctival Lymphoma after Individualized Lens-Sparing Electron Radiotherapy: Results from a Longitudinal Study

**DOI:** 10.3390/cancers15225433

**Published:** 2023-11-15

**Authors:** Christian Hoffmann, Toke Ringbaek, Anja Eckstein, Wolfgang Deya, Alina Santiago, Martin Heintz, Wolfgang Lübcke, Frank Indenkämpen, Wolfgang Sauerwein, Andrea Flühs, Claudia Le Guin, Andreas Huettmann, Julia von Tresckow, Sophia Göricke, Cornelius Deuschl, Sourour Moliavi, Christoph Poettgen, Thomas Gauler, Nika Guberina, Patricia Johansson, Nikolaos Bechrakis, Martin Stuschke, Maja Guberina

**Affiliations:** 1 Department of Radiotherapy, West German Cancer Centre, University Hospital Essen, 45147 Essen, Germanymaja.guberina@uk-essen.de (M.G.); 2 National Center for Tumor Diseases (NCT) West, Campus Essen, 45147 Essen, Germany; 3 Department of Radiotherapy, Medical Physics Section, University Hospital Essen, 45147 Essen, Germany; 4 Department of Ophthalmology, West German Cancer Centre, University Hospital Essen, 45147 Essen, Germany; 5 Department of Hematology, West German Cancer Centre, University Hospital Essen, 45147 Essen, Germany; 6 Institute for Diagnostic and Interventional Radiology and Neuroradiology, University Hospital Essen, 45147 Essen, Germany; 7 Institute of Cell Biology (Cancer Research), Faculty of Medicine, University of Duisburg-Essen, 45147 Essen, Germany; 8 German Cancer Consortium (DKTK), Partner Site University Hospital Essen, 45147 Essen, Germany

**Keywords:** conjunctival lymphoma, ocular adnexal tumors, MALT lymphomas, radiotherapy, electrons, lens sparing, radiation cataracts

## Abstract

**Simple Summary:**

This study investigates whether lens-sparing electron irradiation of low-grade, conjunctival lymphomas prevents cataract formation while ensuring high disease control rates. This study presents the data of 65 eyes of 56 patients with low-grade Ann Arbor stage I conjunctival lymphomas that were treated with either lens-sparing or non-lens-sparing electron irradiation. After a median follow-up of 65 months, the cumulative incidences of 5- and 10-year outfield progression were 10.4% and 13.4% while the cataract incidence was significantly lower in patients treated with a lens-shielding technique. The presented data underline the status of radiotherapy as first line therapy for low-grade conjunctival lymphomas.

**Abstract:**

Irradiation with electrons is the primary treatment regime for localized conjunctival low-grade lymphomas. However, radiation-induced cataracts are a major cause of treatment-related morbidity. This study investigates whether lens-sparing electron irradiation produces sufficient disease control rates while preventing cataract formation. All consecutive patients with strictly conjunctival, low-grade Ann Arbor stage IE lymphoma treated with superficial electron irradiation between 1999 and 2021 at our department were reviewed. A total of 56 patients with 65 treated eyes were enrolled with a median follow-up of 65 months. The median dose was 30.96 Gy. A lens-spearing technique featuring a hanging rod blocking the central beam axis was used in 89.2% of all cases. Cumulative incidences of 5- and 10-year infield recurrences were 4.3% and 14.6%, incidences of 5- and 10-year outfield progression were 10.4% and 13.4%. We used patients with involvement of retroorbital structures treated with whole-orbit photon irradiation without lens protection—of which we reported in a previous study—as a control group. The cumulative cataract incidence for patients treated with electrons and lens protection was significantly lower (*p* = 0.005) when compared to patients irradiated without lens protection. Thus, electrons are an effective treatment option for conjunctival low-grade lymphomas. The presented lens-sparing technique effectively prevents cataract formation.

## 1. Introduction

Indolent non-Hodgkin Lymphomas are among the most frequent histological subtype of primary malignant orbital tumors [1]. Promising data exist underlining the effectiveness and safety of local radiotherapy [2,3,4,5,6,7,8,9,10,11,12,13,14,15,16,17]. Depending on the exact adnexal sub-localization, different treatment techniques may be applied [18,19]. At this department, lymphomas that are restricted to the conjunctiva are routinely treated with en face electrons. Irradiation of the whole-orbit with photons is used in case of intraorbital tumor spread or whenever combined conjunctival and intraorbital involvement occurs [20,21].

Previous publications most often do not differentiate between “orbital-type” lymphomas treated with photon-beams and “strictly conjunctival” lymphomas treated with electron beams with limited range. The technical properties differ significantly and should be strongly considered in terms of outcome and side effects. The therapeutic dose distribution of electron beams is restricted to the anterior third of the orbit and lens-sparing techniques that block the central beam axis providing the lens sparing. Therefore, the side effects might differ between the photon and electron treatment techniques.

In previous studies, local failures solely or with a high proportion occurred in patients treated with electrons [2,11,12,13,17,22]. Small electron fields for conjunctival lymphomas are a challenging treatment technique that involves multiple components such as lens-shielding rods, superflaps, individualized collimators and immobilization devices. Slight setup errors lead to biologically significant underdosing. We therefore put special emphasis on creating a homogenous cohort of indolent, strictly “conjunctival-type” lymphomas with Ann Arbor stage IE. To quantify radiotherapy related side effects, we applied the CTCAE criteria and evaluated changes in visual acuity. We used a previously published cohort of orbital-type lymphoma patients that were treated with non-lens-sparing whole-orbit irradiation as a control group [21].

## 2. Methods

### 2.1. Patients

We retrospectively analyzed all patients diagnosed with strictly conjunctival, low-grade lymphomas that were treated between 1999 and 2021 at the Department for Radiotherapy of the University Hospital Essen. Only patients with histologically confirmed Ann Arbor stage IE were enrolled (Table 1). The initial staging procedures included an abdominal and thoracic CT scan, a bone marrow biopsy, a cranial MRI and a peripheral blood analysis. All patients were discussed in our internal, multidisciplinary cancer conference. We did not administer Rituximab concomitant with radiotherapy. Three patients were initially treated with Rituximab but did not sufficiently respond or had progressive disease. In six patients, unsuccessful first-line antibiotic therapy was performed prior to irradiation.

We used a previously published cohort consisting of patients with stage I, “orbital-type”, low-grade lymphomas that were uniformly treated with whole-orbit photon irradiation as the control group concerning outfield recurrences and cataract formation. These patients were treated with a non-lens-shielding technique and a median dose of 30.6 Gy [21].

Institutional review board approval was obtained at the University Hospital Essen to conduct this retrospective study, and informed consent was waived (Ethics Committee of the Medical Faculty of the University Duisburg-Essen, ID 21-10036-BO) as only anonymized data were used. All procedures were performed according to the Declaration of Helsinki’s relevant guidelines and regulations.

### 2.2. Follow-Up and Evaluation Criteria

Patients underwent slit-lamp examinations once to twice per year by an ophthalmologist and were offered structured multidisciplinary follow-up examinations by an haemato-oncologist and a radiation oncologist. Radiological follow-up procedures were only performed on suspicion of recurrent disease.

Acute and late toxicities were evaluated according to the Common Terminology Criteria for Adverse Events (CTCAE) version 5.0. The visual acuity was assessed by a decimal chart. Decimal scores were then converted with the following formula: logMAR = −log(decimal acuity) [23]. We compared the following time points: before irradiation, 0–12 months, 12–24 months and more than 24 months after treatment.

### 2.3. Treatment Techniques

Irradiation was performed by use of single beam, en face, 6–12 MeV electrons (Table 2). We routinely use round 5.0 or 5.5 cm electron collimators applicators with a source to skin distance of 100 cm. An optimal field shape was achieved with a made-to-measure cerrobend block fixed to the distal part of the collimator. Since 2011, we treated most patients with 6 MeV electrons. Total doses ranged from 25.2 to 34.4 Gy with a median dose of 31.0 Gy and a mean dose of 29.7 Gy.

Until March 2019, the dose was prescribed according to open field calculations and standardized procedures estimating the overall collimator effects. Thereafter, reference dosimetry was established using phantom measurements. Depth dose distributions were measured in a water phantom with a lens block and individualized collimators infield. The dose prescription point was set to be in the dose plateau region around the lens-shielding rod at a depth directly below the bolus material [24]. Since then, standard dose prescription was 25.2–28.8 Gy (1.8 Gy/fraction). Prescribed doses applied before March 2019 were converted to those after that date (Table 2).

**Figure 1:**
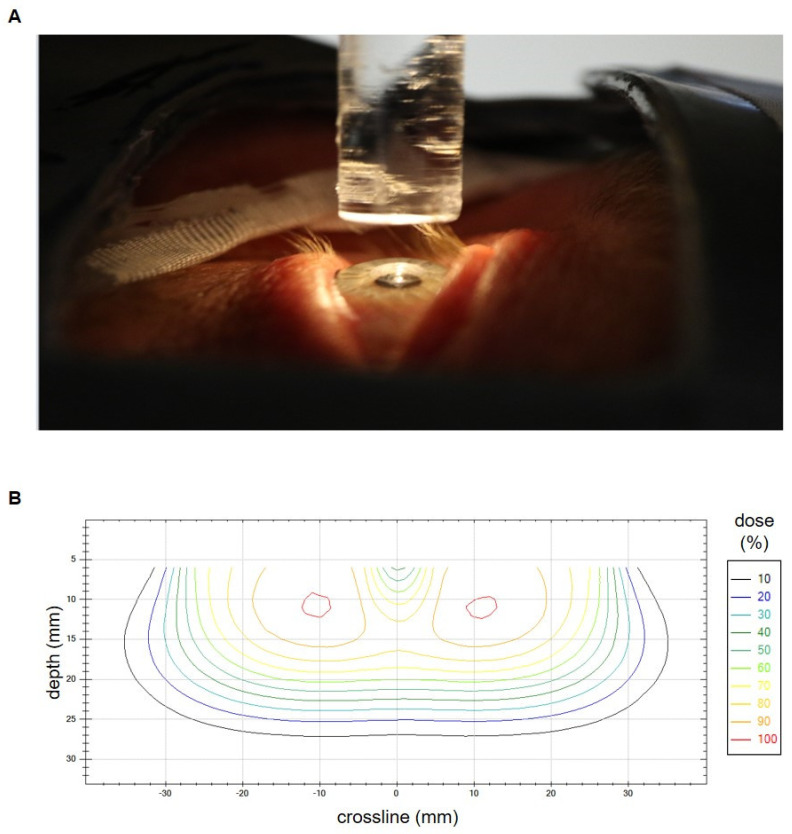
(**A**) Setup for conjunctival irradiation with electrons and lens shielding just before the bolus application. The lens-shielding rod is mounted to the distal part of the electron applicator and spares the central beam axis. It is fixed to an acrylic glass plate and is additionally equipped with a light-emitting diode to facilitate the patients’ fixation of its central part. An additional bolus is used in case of superficial tumor spread. Mask fixation and lid retraction with plasters allow for an optimal immobilization. A lead shield covers the non-target regions. (**B**) Isodose crossline resulting from in-house measurements of a typical lens-sparing treatment setup with 6 MeV electrons, a round 55 mm tertiary aperture and a 7 mm (width) × 70 mm (length) lens-shielding rod. The measurement was performed on a Varian Clinac 2100 C/D linear accelerator. The lens in the central beam axis is spared.

To achieve optimal lens sparing, we routinely used a lens-shielding rod to block the central-beam axis that was attached to an acrylic glass plate. The rod diameter was chosen accordingly to the patient’s eye anatomy. The used diameters ranged from 7 mm to 10 mm and the corresponding length ranged from 40 mm to 70 mm. Seven eyes (10.8% of all eyes) were irradiated without lens protection. To allow for proper eye fixation in the center line, a light-emitting diode was placed above the pin. An additional superflap bolus of 3 mm to 10 mm, or a liquid bolus (HPMC gel) directly applied to the eye, was used to improve dose coverage of the anterior part of the target volume. Immobilization was performed by standard via mask fixation except for cases in the early 2000s. Figure 1A shows a typical treatment setup. Figure 1B depicts an isodose crossline resulting from in-house measurements of a typical lens-sparing treatment setup with 6 MeV electrons, a round 55 mm tertiary aperture and a 7 mm (width) × 70 mm (length) lens-shielding rod. The measurement was performed on a Varian Clinac 2100 C/D linear accelerator by use of a waterphantom (Blue Phantom, IBA Dosimetry GmbH, Schwarzenbruck, Germany) and a diamond detector (SN 6-022, PTW Freiburg GmbH, Freiburg, Germany).

The EQD2 was calculated as follows:

D_2Gy_/D_given_ = (α/β + d_given_)/(α/β + d_2Gy_).

We used an α/β-ratio of 3 for organs at risk and of 10 for lymphomas [2].

All toxicities were graded according to the CTCAE criteria (Version 5).

### 2.4. Statistical Analysis

SPSS Statistics 27.0 software (IBM) or SAS (version 14.1, SAS Institute) were used for statistical analysis. Freedom from progression was analyzed by the Kaplan–Meier method using time to progression at any site as events and time of last follow-up as censoring events for patients without relapses. Progression-free survival curves were compared by the log-rank test. Cumulative incidences of infield recurrences and outfield relapses were determined using infield and outfield relapses as concurrent risks. Data were censored at the last clinical follow-up. Gray’s test was used to assess equality of cause-specific cumulative incidences. We performed the competing risk analysis for in- and outfield progression per treated eye rather than per treated patient.

A small proportion of patients initially presented with bilateral disease were treated simultaneously. Patients with bilateral conjunctival lymphoma had a greater risk of outfield recurrences than patients with unilateral lymphoma, which suggests that both eyes with conjunctival involvement act independently on the risk of distant relapse. However, analyzing the risk of outfield relapses per treated eye, the risk of outfield relapses was similar for unilateral and synchronous bilateral disease. If an outfield occurred in a patient with synchronous bilateral disease, the event was counted for one eye while the observation of relapses from the other eye was censored just before the occurrence of the outfield relapse. Cumulative incidences of cataracts were calculated using the last clinical follow-up as censoring events. The unpaired *t*-test was used to analyze changes in visual acuity. 

## 3. Results

### 3.1. Local Effectiveness

**Figure 2:**
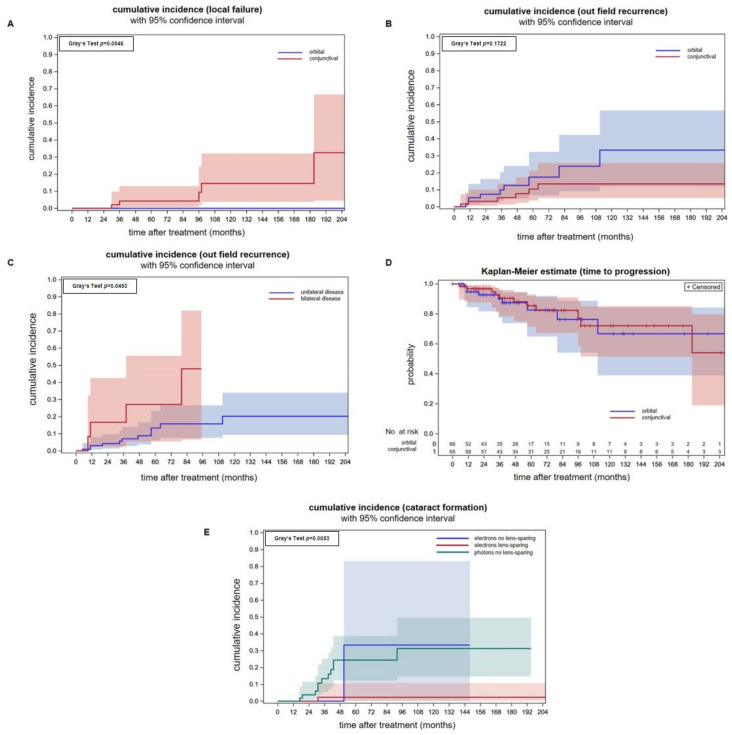
(**A**) Cumulative incidences of infield recurrences with 95% confidence intervals for individual predictions for conjunctival lymphomas and superficial electron beam treatment (red line) and orbital lymphomas with whole-orbit photon irradiation (blue line). Analysis was performed per involved ocular adnexa. (**B**) Cumulative incidences of outfield recurrences per involved ocular adnexa are not significantly different between conjunctival lymphomas treated with electrons and orbital lymphomas treated with a photon technique. (**C**) Cumulative incidences of outfield recurrence in a per patient analysis showing a significantly higher incidence in patients that initially presented with bilateral tumor spread (Gray’s test *p* = 0.045). In this analysis, patients with orbital and conjunctival lymphomas were included. (**D**) Freedom from progression of conjunctival lymphomas after electron therapy in an analysis per involved ocular adnexa. There were no differences in comparison to orbital lymphomas treated with electrons. (**E**) The cumulative cataract incidences in patients treated with a lens-sparing approach (red line) is significantly lower (Gray’s test *p* = 0.005) when compared to patients treated with a non-lens-sparing approach (green and blue line). Seven eyes were treated with electrons without lens sparing (blue line). Orbital-type lymphoma patients treated with non-lens-sparing whole-orbit photon irradiation (green line) are plotted as a control group. The applied median doses were comparable (conjunctival-type: 31 Gy, orbital-type: 30.6 Gy).

In all, 56 patients met the inclusion criteria, and 65 eyes were irradiated (Table 1). The predominant histological subtype was MALT lymphomas. The median follow-up was 65 months. Cumulative incidences of infield recurrences were 4.3% (95% CI: 0.8–13.0%) and 14.6% (95% CI: 3.8–32.1%) after 5- and 10-years. All infield relapses were restricted to the conjunctiva as radiological staging procedures showed no retroorbital disease spread. All patients with infield relapse were treated before 2010. We compared the above data with a previously published patient cohort consisting of orbital-type lymphoma patients that were uniformly irradiated with whole-orbit photon irradiation using a non-lens-sparing approach. The applied median doses for both groups were comparable (conjunctival-type: 31.0 Gy, orbital-type: 30.6 Gy) (Figure 2A) [21]. No local failure occurred in the photon group but the difference between the cumulative incidence curves were not significant (Gray’s test *p* = 0.0546). Patients with infield recurrences were either treated with Rituximab or a watchful waiting strategy due to their advanced age. 

We continuously improved the presented treatment technique during the observation period. As a major improvement, rigid fixation methods such as mask fixation and lid retraction with plasters or retractors were introduced. Cox regression analysis of prognostic factors for infield relapses revealed the use of a rigid mask fixation to be an important factor for local control with an associated hazard ratio of 9.32 (95%CI: 0.97–98.4; *p* = 0.0529; chi2 test). 

### 3.2. Outfield Progression

The cumulative incidences of outfield progression at 5- and 10-years were 10.4% (95% CI: 3.6–21.3%) and 13.4% (95% CI: 5.2–25.7%) after electron radiotherapy of conjunctival lymphoma on a per eye analysis (Figure 2B). Outfield progression occurred in the majority of cases in the contralateral eye. Two patients with isolated relapse in the contralateral eye were irradiated successfully. The remaining patients received Rituximab in combination with Bendamustin. We compared the above data with a previously published patient cohort consisting of orbital-type lymphoma patients treated with photon radiotherapy [21]. There was no statistically significant difference between the cumulative incidences of outfield recurrences between both tumor localizations, although the incidences were numerically higher for orbital lymphomas Gray’s test *p* = 0.172). Patients treated with photons had a cumulative incidence of outfield recurrences of 17.4% (CI: 6.7–32.3%) and 33.3% (95% CI: 11.4–56.6%) at 5- and 10-years. Analyzing outfield recurrences of conjunctival and orbital lymphoma together on a per patient basis, a higher incidence of patients with synchronous bilateral lymphomas was found (*p* = 0.0453, Gray’s test, Figure 2C). This is consistent with the idea that both involved eyes act independently on the incidence of distant recurrence. In and per ocular adnexa analysis, however, the cumulative incidences of outfield relapses were similar (*p* = 0.4498, Gray’s test).

The freedom from progression of the group of patients with conjunctival lymphomas at 5-,10- and 15-years was 85.4% (95% CI: 71.4–92.8%), 72.0% (95% CI: 51.4–85.1%) and 72.0% (95% CI: 51.4–85.1%). There is no difference in freedom from progression between conjunctival and orbital lymphomas (*p* = 0.02, log-rank test, Figure 2D).

### 3.3. Acute and Late Toxicities

The predominant acute toxicities were conjunctival irritations (crude incidence: 60%) and local dermatitis (41.5%) that all were CTCAE grade 1 (Table 3).

The most common late toxicities were dry eyes (crude incidence: 13.8%) and conjunctival irritations (6.2%) of which none was higher than CTCAE grade 1. Dry eye symptoms had no negative effect on visual acuity and only required stage 1 therapy.

Treatment-related cataracts occurred in two patients (3.4% of all eyes at risk). Patients with intraocular lens replacement before irradiation were excluded from the analysis (n = 7 eyes). One patient underwent cataract surgery (CTCAE grade 2) while the other patient did not require treatment and suffered only from a minor decrease in visual acuity (CTCAE grade 1). 

Figure 2E shows that the lens-sparing approach effectively prevents cataract formation. The cumulative cataract incidence for patients treated with electrons and lens protection was significantly smaller (Gray’s test, *p* = 0.005) when compared to patients treated with electrons without a lens-sparing technique or photons for orbital lymphoma. Electrons without lens sparing were used in a minority of patients (n = 7 eyes).

### 3.4. Visual Acuity before and after Treatment

**Figure 3:**
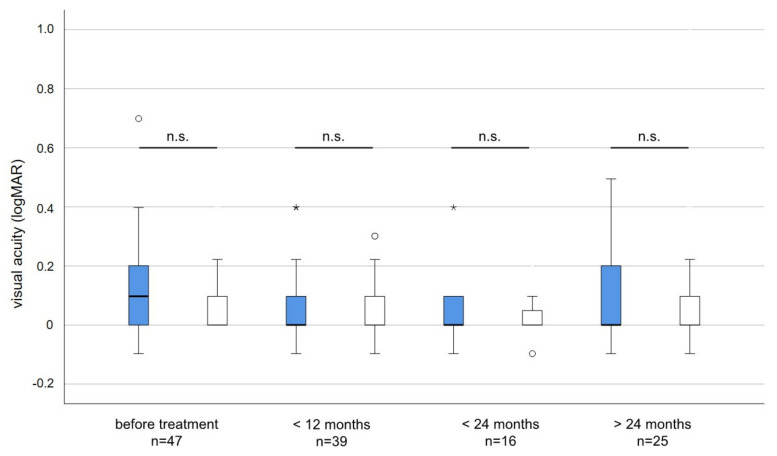
Visual acuity (logMAR) of irradiated (blue) and non-irradiated eyes (white) before and after treatment. The average visual acuity before irradiation was slightly but not significantly worse in the affected when compared to the unaffected eye (Unpaired *t*-test *p* = 0.084). No statistically significant changes or differences in visual acuity occurred at any time point during follow-up. n.s. = not significant, * = outlier > three times interquartile range. circles = outlier more than 1.5 times the interquartile range.

The average visual acuity before irradiation was slightly but not significantly worse (logMAR 0.12 versus logMAR 0.07; *p* = 0.084) in the affected compared to the non-affected eye. During long-term follow-up, the average visual acuity did not deteriorate and differences in visual acuity between treated and untreated eyes were neglectable and not statistically significant (Figure 3).

## 4. Discussion

This single-institutional study analyzes the long-term outcomes of 56 patients with localized, “conjunctival-type”, low-grade stage IE lymphomas treated with electrons. Patients with orbital involvement were typically treated with whole-orbit irradiation using photons. Results with that technique were previously reported and used for comparison [21].

Our data strengthen the role of electron radiotherapy as the primary treatment regime for conjunctival low-grade lymphomas. The 5-year incidence of local recurrences and freedom from progression were 4.3% and 85.4%, which were in line with the previously published data on progression-free survival (5-year PFS range: 75–100%) [2,4,7,8,9,10,11,16,17]. After 2010, introducing routinely fixed masks local control rates was 100%. However, in previous studies that collectively analyzed patients treated with photons and electrons, local failures predominantly occurred in patients treated with electrons [2,11,12,13,17,22]. Small electrons fields were designed to deliver homogeneous doses to the superficially located conjunctiva while creating a steep dose gradient to the lens and the posterior globe. This was achieved by use of a lens-sparing approach and the application of individualized secondary and tertiary lead collimators [25]. The use of a rigid mask fixation is an important factor for local control.

The electron energies used in previous studies ranged from 3 to 20 MeV [2,5,6,10,11,12,13,16,17]. Chow et al. dosimetrically analyzed the effect of 4, 9 and 16 MeV on the lens-shielding efficiency and pointed out that increasing energies negatively influence the shielding efficiency due to increased side scattering and an increased penumbra width [26]. It is noteworthy that Chow et al. used a shielding lens directly attached to the cornea in contrast to the hanging rod used in this study. Brualla et al. directly compared 6 to 9 MeV for the exact setup used in our cohort and found that 6 MeV provides a sufficient dose coverage of the target volume but allows for better sparing of the posterior orbit than 9 MeV [25]. Higher energies (e.g., 16 MeV) cause unnecessary dose exposure to posterior orbital structures [27]. With regard to a bolus, a 7 mm hole in the bolus did not significantly alter the dose distributions in the study by Young et al. but allowed the patient to focus on the hanging lens shield and might thus improve the daily reproducibility [28]. Given the before-mentioned data 6, MeV is sufficient for most cases. 

In principle, two types of lens-sparing devices exist: a contact lens with a mounted lead block or a hanging rod attached to the distal part of the electron applicator [2,4,5,6,10,11,12,16,17,22,25,26,28,29]. The contact lens type immobilization techniques might cause discomfort to the patient but the lack of a shield-to-surface distance has dosimetric advantages [28,30,31]. Increasing shield-to-surface distance goes along with increased lens doses due to lateral electron scattering into the air gap. Borger and Rustgi et al. state that the distance should be kept below 1 cm [29,31]. In this cohort, we observed treatment-related cataracts in two patients (3.4% of all eyes at risk), which is in line with previously published data [2,22]. The present study shows that lens sparing can significantly reduce the incidence of cataracts for patients with conjunctival lymphomas.

There is a dose–response relationship for follicular lymphomas in different sites as shown by a randomized trial comparing low dose irradiation at a total dose of 4 Gy with moderate dose irradiation at 24 Gy given with conventional fractionation. Local control rates at 5 years were 88% at 24 Gy and 67% at 4 Gy (*p* < 0.0001) [32]. Imber et al. report 2-year local progression rates of 9% for localized indolent lymphomas treated with 4 Gy [33]. The high control rates in previous studies on orbital lymphomas giving total doses of about 30 Gy with conventional fractionation as well the results of the present study support that a moderate dose reduction with conventional fractionation might be performed using high precision electron therapy [2,4,5,6,10,11,12,13,15,16,17]. As toxicity of the lens-sparing technique is low, a careful consideration of an increased risk of relapse and further reduction in the toxicity is necessary. Long-term complications of grade III or higher did not occur in our cohort in contrast to the reports by McGrath et al. in a review article [34]. Visual acuity did not significantly deteriorate after treatment in the present cohort. A total dose of 24–30 Gy with conventional fractionation is considered as standard according to the most recent NCCN guidelines.

Some studies propose alternative front-up treatment regimens such as Rituximab monotherapy or first-line chemotherapy. Most of them are limited in terms of patient numbers and follow-up periods. Ferreri and colleagues published the data of 20 conjunctival-type, MALT lymphoma patients treated with intralesional Rituximab monotherapy. At a median follow up of 42 months, they reported a 5-year PFS rate of 59% [35]. Another study compared upfront radiotherapy with intravenous Rituximab monotherapy for both conjunctival and orbital type MALT lymphomas. After a median follow up of 48.8 months, the 5-year PFS rate in the Rituximab group (n = 19) was 41.4% compared to 67.4% in the radiotherapy group (n = 24) [36]. A large multicenter, retrospective cohort study showed a significantly better 10-year disease specific survival for stage I ocular MALT lymphoma patients when treated with radiotherapy rather than systemic treatment. Outcomes for systemic treatment were significantly better for patients treated with chemotherapy containing Rituximab than for patients treated with chemotherapy without Rituximab. However, the informative value of this data is reduced due to the heterogeneity of the applied systemic treatment regimes [37]. Two small studies with CVP-based systemic treatment showed inferior PFS rates than we observed in the present conjunctival-type cohort or in a previously published orbital-type cohort [21,38,39].

In Table 4, we summarize major studies on this subject published in the past 10 years (Table 4). With modern planning devices and techniques, control of conjunctiva lymphoma can be achieved alongside with minimizing persistent and higher grades of radiation side effects.

## 5. Conclusions

This monocentric study shows that the lens-sparing electron-radiotherapy of “conjunctival-type”, low-grade non-Hodgkin lymphomas results in high local control rates. The treatment is well tolerated and long-term toxicities are mild. The presented lens-sparing technique effectively reduces the cataract formation incidence. We achieved high control rates at median doses of 31 Gy. Hence, our data support the current trend to use slightly lower total doses with conventional fractionation.

## Figures and Tables

**Table 1:** Patient characteristics.

Characteristic	No. of Patients/Value
Age at diagnosis (years)	
Median	54
Range	27–78
Follow-up (months)	
Median	65
Range	6–280
Sex	
Male	26
Female	30
Number of irradiated eyes	65
Bilateral disease	8
Histological subtype	
MALT	51
Follicular lymphoma (grade 1–2)	9
Lymphoplasmacytic lymphoma	3
Mantle cell lymphoma	2

**Table 2:** Treatment characteristics.

Characteristic	No. of Eyes/Value
Technical specifications	
Lens-sparing rod	58
HPMC eye-drop bolus	45
Superflap bolus	27
Radiotherapy dose (Gy; Gy/F)	
Median dose per fraction (range)	1.72 (1.6–1.8)
Median total dose (range)	31 (25.2–34.4)
Median total dose as EQD2 (a/b = 3 Gy) (range)	28.2 (24–32.5)
Median total dose as EQD2 (a/b = 10 Gy) (range)	29.8 (24.8–33.6)
Energy (MeV)	
6	47
9	11
6 + 9	5
12	2
Immobilization	
Mask + lid fixation	46
Mask fixation only	9
No fixation	6
Lid fixation only	4

Applied doses are specified in Gray (Gy) and Gray per fraction (Gy/F). Prescribed doses applied before March 2019 were converted as described in the Methods section. A hanging rod blocking the central beam axis was used for lens sparing. A hydroxypropyl methylcellulose (HPMC) eye-drop bolus was routinely applied to avoid underdosing to the anterior part of the target volume. In rare cases, a combination of 6 and 9 MeV electron beams was used consecutively.

**Table 3:** Acute and late toxicities according to the CTCAE v5.0 criteria.

Early Toxicities	Grade 1	Grade 2	Grade 3
Conjunctivitis	39 (60%)	0	0
Dermatitis	27 (41.5%)	0	0
Watering eyes	13 (20%)	0	0
Pain	0	0	0
Periorbital edema	0	0	0
**Late toxicities**			
Dry eyes	9 (13.8%)	0	0
Conjunctival irritation	4 (6.2%)	0	0
Watering eyes	3 (4.6%)	0	0
Unilateral cataract	1 (1.7% *)	1 (1.7% *)	0
Keratitis	2 (3.1%)	0	0
Maculopathy	0	0	0
Optic neuropathy	0	0	0

In total, 65 eyes were irradiated in 56 patients. * Patients with intraocular lens replacement before irradiation were excluded from the cataract analysis (n = 7 eyes).

**Table 4:** Publications from 2013 to 2023 including patients with conjunctival lymphoma and electron radiotherapy.

Publication	Affiliation	Publication Year	Years of Treatment	Pts. [n]	Eyes [n]	Radiation Therapy Technique	Radiation Dose [Range (Median) [Gy]]	Dose per Fraction [Gy]	Cataract [%]	Follow-Up Time [Range (Median) [Months]]	Local Control Rate [%]
Harada et al. [17]	Tokyo, and Chiba, Japan	2014	1990–2010	86	104	X-rays 63 eyes; E 41 eyes	30.0–46.0 (30)	2.0	55.38 without lens- shielding 30.77 with lens- shielding	10.8- 264 (108)	99.14
Woolf et al. [19]	London, UK	2014/ 2015	2002–2012	81	85	kV technique; 20 eyes MV technique 65 eyes	30.0–35.0 (30.0)	2.0	8	2.4–124.8 (52.8)	100
Parikh et al. [4]	New York, USA	2015	1995–2012	79	85	E, 3D or IMRT	21.0–36.0 (30.6)	1.5–1.8	14	(median 49.7)	100
Desai et al. [8]	Miami, FL, and Standford, CA, USA	2017	1984–2015	182	196	conjunctival lymphoma were treated mainly with E	22.0–45.0 (30.6)	1.8–2.0	Low toxicity	2–387 (63)	97.0 (Ann Arbor stage I)
Platt et al. [15]	Cleveland, Ohio, USA	2017	1997–2015	60	77	E or IGRT	20.0–36.0	1.5–2.0	25	0–194 (38)	100
Park et al. [18]	Seoul, Korea	2017	2001–2016	67	76	Photons: 10 pts; E 57 pts	22.0–45.0 (30.0)	1.8–2.0	26.87	1.9–149.4 (50.9)	100
Lee et al. [22]	Seoul, Korea	2019	1993–2013	212	246	Photons 80 pts E 132 pts	16.2–36.0 (25.2)	1.8–2.0	10.98	3–271 (70)	91.4
Olsen et al. [40]	Multicenter study	2019	1980–2017	797	915	n.s.	4.0–40.0 (26)	n.s.	n.s.	0–399 (35)	86.2
Hindsø et al. [37]	Multicenter study	2020	1980–2017	689	791	photons	4.0–60.0 (26)	n.s.	n.s.	0–438 (42)	81.9
Rehn et al. [9]	Muenster, Germany	2020	2003– 2019	45	52	Photons: 39 eyes E: 13 eyes	4.0–50.4 (36)	0.5–2.0 (median 1.8)	rated as most frequent adverse event	2–170 (33)	100
MacManus et al. [41]	Multicenter prospective trial	2021	2006–2014	70	39	MV-Photons or E	24.0–30.6 (n.s.)	1.5–2.0	71.1	8.4–112.8 (60)	98.6
Yang et al. [42]	Shanghai, China	2022	2019–2021	16	21	n.s.	4.0 (4)	2.0	n.s.	5.0–30.0 (15.5)	85.0
Hoffmann et al./study results 2023	Essen, Germany	2023	1999–2021	56	65	E (89.2% lens-sparing technique)	25.2–34.4 (31.0)	1.6–1.8 (1.72)	3.4	6–280 (65)	Including pts. before 2010: 85.4. All pts. with rigid mask systems after 2010: 100

Pts.: Patients; E = Electrons; IGRT = Image-guided intensity-modulated radiation therapy, EBRT = external beam radiation therapy; IMRT = intensity-modulated radiation therapy; 3D CRT = three-dimensional conformal radiation therapy; VMAT= volumetric-modulated arc therapy; n.s. = not specified.

## Data Availability

The data of this study were collected during regular clinical care and can therefore not be shared.
